# Numerical Analysis of a Transtibial Prosthesis Socket Using 3D-Printed Bio-Based PLA

**DOI:** 10.3390/ma16051985

**Published:** 2023-02-28

**Authors:** Vasja Plesec, Jani Humar, Polona Dobnik-Dubrovski, Gregor Harih

**Affiliations:** 1 Laboratory for Intelligent CAD Systems, Faculty of Mechanical Engineering, University of Maribor, Smetanova ulica 17, 2000 Maribor, Slovenia; 2 Mechanical Engineering Research Institute, Faculty of Mechanical Engineering, University of Maribor, Smetanova ulica 17, 2000 Maribor, Slovenia

**Keywords:** 3D-printing, bio-based, polylactic acid, PLA, prosthesis, prosthetic socket, numerical model, finite element method

## Abstract

Lower-limb prosthesis design and manufacturing still rely mostly on the workshop process of trial-and-error using expensive unrecyclable composite materials, resulting in time-consuming, material-wasting, and, ultimately, expensive prostheses. Therefore, we investigated the possibility of utilizing Fused Deposition Modeling 3D-printing technology with inexpensive bio-based and bio-degradable Polylactic Acid (PLA) material for prosthesis socket development and manufacturing. The safety and stability of the proposed 3D-printed PLA socket were analyzed using a recently developed generic transtibial numeric model, with boundary conditions of donning and newly developed realistic gait cycle phases of a heel strike and forefoot loading according to ISO 10328. The material properties of the 3D-printed PLA were determined using uniaxial tensile and compression tests on transverse and longitudinal samples. Numerical simulations with all boundary conditions were performed for the 3D-printed PLA and traditional polystyrene check and definitive composite socket. The results showed that the 3D-printed PLA socket withstands the occurring von-Mises stresses of 5.4 MPa and 10.8 MPa under heel strike and push-off gait conditions, respectively. Furthermore, the maximum deformations observed in the 3D-printed PLA socket of 0.74 mm and 2.66 mm were similar to the check socket deformations of 0.67 mm and 2.52 mm during heel strike and push-off, respectively, hence providing the same stability for the amputees. We have shown that an inexpensive, bio-based, and bio-degradable PLA material can be considered for manufacturing the lower-limb prosthesis, resulting in an environmentally friendly and inexpensive solution.

## 1. Introduction

Due to injury-induced accidents or diseases, a significant number of people are losing limbs, notably legs. It has been shown this is a life-changing experience affecting the patient’s ability to move, work, socialize, and maintain independence [1]. Hence, restoring the mobility of a patient after leg amputation has been shown to be one of the most important aspects of successful reintegration in social life. Longer life expectancy and an increase in age-related diseases (blood vessel diseases, cancer, infections, tissue damage, etc.), among which diabetes is the most prominent, have led to a steady increase in nontraumatic leg amputations [2]. It is estimated that around 150,000 leg amputations are performed every year in the United States, mostly as a result of diabetes complications, with an increased rate of 50% from 2009 to 2015 [3].

Patient recovery after limb amputation is complicated and long lasting, usually never reaching the life quality prior to amputation. To minimize the immediate and long-lasting detrimental effects of amputations, patients usually get prostheses designed and manufactured by hospitals and rehabilitation centers for the restoration of mobility and, hence, successful reintegration in social life [4]. The advancement of understanding of the biomechanics of the prosthesis–limb system and lower-limb prosthesis design and manufacturing technologies has led to measurable improvements in prosthesis fit, pain reduction, reduction in tissue and joint loading, metabolic cost, gait improvement, and even appearance [5,6,7,8,9,10].

Despite the presented advancements, significant numbers of patients still report low satisfaction levels due to the poor fit between the prosthesis and the residual limb [11]. Prosthetists still rely on their knowledge, experience, and craftsman skills to design and manufacture a prosthesis socket for a specific patient [4]. An additional problem presents as the hard-to-communicate subjective feeling of the test prosthesis fit (comfort, stability, etc.). This workshop process of trial-and-error, with frequent prosthesis changes and with high costs of materials, has shown to be time-consuming and material-wasting, and, ultimately, expensive [12,13]. Previous research has shown that the manufacturing of patient-specific prosthesis sockets in the first five years after amputation ranges from approx. USD 6000 to almost USD 20,000 [14].

Therefore, the prosthesis-socket–limb interface fit still presents numerous challenges and, hence, opportunities for improvement. Recent advancements in integrated design and manufacturing processes and computer-aided technologies (CAD/CAM/CAE) and 3D-printing present great possibilities to manufacture cost-efficient and functional products [12,13,15]. In prosthetics, the processes and methods of manufacturing prostheses are currently under development using 3D-printing, which requires obtaining the stump’s geometry using various technologies (3D scanner, CT scan, MRI, etc.), virtual rectification inside computer software, prosthesis design, and 3D-printing of the prosthesis [16,17]. While the prosthesis computer-integrated design process with 3D-printing manufacturing technology shows great potential, the usage in clinical practice is still on a small scale due to the lack of a systematic framework that would integrate all technologies in a seamless prosthesis development and manufacturing solution [12].

Stereolithography (SL), Selective Laser Sintering (SLS), and Fused Deposition Modeling (FDM) have already been investigated for the fabrication of lower-limb prosthetic sockets, and have shown promising results [18,19,20,21]. Although rapid prototyping methods differ significantly in terms of cost, performance, available materials, etc., they are all based on the principle of building parts by adding thin layers in horizontal series. The mechanical properties of the final printed part can be enhanced by adjusting the 3D-printing process variables, including printing speed, nozzle temperature, printing strategy, and other factors [22,23]. With the rapid evolution of additive manufacturing and the development of new printable materials, 3D-printed parts can be used not only as prototypes, but also as end products. Furthermore, conventional sockets can be improved in terms of fit and comfort with the additive manufacturing process by designing different areas of compliance for the reduction in stress concentration [24,25]. A recent observational cohort study investigated low-cost 3D-printed transtibial prostheses made from PLA filament using the FDM process [17]. The prostheses were evaluated in a rural population in Sierra Leone, and the results showed the safety and functionality of 3D-printed prostheses. PLA is the most widely used plastic filament in the FDM 3D-printing, as it shows good printability, it is biodegradable, and it can be produced economically from renewable resources. However, PLA has not been used widely as an engineering plastic, as it shows relatively lower strength, thermal, chemical, and/or impact resistance, compared to other more established engineering plastics, such as polyamides, polycarbonates, and acrylonitrile butadiene styrene, not to mention plastic composites such as glass or carbon-reinforced plastics [26]. It has been shown that the main problem of 3D-printed parts using FDM printing technology is layer delamination due to bad layer-to-layer adhesion; therefore, extensive material testing needs to be performed in various directions to obtain accurate material properties for product design and dimensioning [17]. The strength of the 3D-printed parts is dependent on the resistance of the filaments, the resistance of the union between filaments of the same layer, and the resistance of the union between layers [27]. Despite showing great potential, functional products such as 3D-printed PLA sockets still need to be tested numerically and experimentally to prove their applicability throughout the life cycle of the prosthesis.

It has been shown that the well-established Finite Element Methods (FEM) can be used successfully in prosthetics and orthotics to test and evaluate medical devices numerically [28]. Numerical simulations can accelerate the development of a new prosthetic component, such as a liner or socket, and reduce the number of physical prototypes needed. By analyzing the results in terms of numerical stresses, strains, contact pressures, etc., which are otherwise difficult to obtain with physical tests, one can evaluate multiple designs and materials effectively in a virtual environment. Despite recent advances in computer-aided technologies, the results should be evaluated with caution due to numerical limitations. For example, the material models used to model biological tissue represent only a time-independent approximation, and the geometry of the residual limb does not account for volume variations. Therefore, numerical results obtained with different socket-liner designs or materials should be compared relative to each other, rather than evaluating a specific prosthesis for a given lower-limb amputee in absolute terms.

However, most current residual limb models are based on a specific geometry determined by MRI, CT, X-ray, or 3D scanners [29]. While such simulations can forecast the interaction between the given limb and the prosthesis, they cannot be used to analyze the general population, due to the specific geometry. On the other hand, a generic limb model can address a broader group of amputees and serves as a numerical tool for developing new rectification designs and testing prospective prosthetic component materials in a virtual environment [30]. Therefore, a generic model is preferable for the initial evaluation. To date, only a handful of generic models have been developed that attempt to capture the biomechanical interaction representatively between the residual limb and the prosthesis [31,32,33]. One of the more advanced generic models was developed in a recent study. It shows the applicability for predicting the biomechanical interaction in various stages of use: donning of the socket, single-leg stance, heel strike, and push-off for an average male transtibial limb [34].

Different loading cases should be analyzed in order to evaluate the feasibility of prosthetic components such as sockets and liners. The most common loading conditions studied in numerical simulations are donning of the socket and the single- or double-leg stance [28]. While they are useful for determining the initial stresses in the residual limb, they do not predict the stresses that occur during walking. The static instances of the gait should also be assessed to address this issue. To the best of the authors’ knowledge, there is no specific Standard for the testing procedure of prosthetic sockets, and, therefore, no specific loading case for socket evaluation. The closest Standard is SIST EN ISO 10328:2016 Prosthetics—Structural testing of lower-limb prostheses—Requirements and test methods. The Standard is not intended for socket evaluation but rather for other prosthetic components such as knees, feet, and ankles. However, in the absence of more specific Standards or guidelines, the aforementioned Standard is used frequently to assess sockets [17,29,35,36,37]. The forces and moments for heel strike and push-off can be extracted from the Standard and incorporated into the simulation to test the prosthesis numerically at these two critical moments of the gait cycle. A major advantage of the numerical method for socket-liner systems’ assessment is that, by using hyperelastic material models, a nonlinear response of the bulk soft tissue can be obtained, and, thus, a more realistic behavior. In contrast, the foam and rigid plaster of Paris or similar materials used commonly to fill the socket do not reflect the soft tissue response adequately in experimental tests [38,39].

Due to the lack of experimental test procedures for the prosthesis socket evaluation and analysis and the advantages of numerical models as presented in the introduction, the aim of the study was to utilize the recently developed generic numerical model of a transtibial-limb–socket system, and simulate realistic loading scenarios for the evaluation and analysis of the different socket materials. The numerical model was utilized further to evaluate and analyze if a 3D-printed bio-based PLA transtibial socket can withstand the mechanical loads occurring in the socket during the prosthesis’ use. Biomechanical analysis has been performed further on the numerical results, and the 3D-printed PLA socket has been compared to the Polystyrene (PS) check and a definitive composite socket in terms of prosthesis stability.

## 2. Materials and Methods

### 2.1. Testing of 3D-Printed PLA Specimen’s Experimental Material Properties

**Figure 1:**
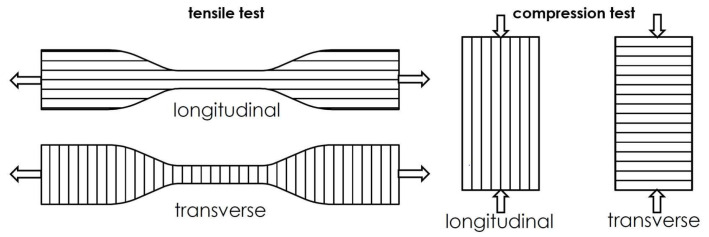
**Left**: Direction of printing for the Type IV specimen according to Standard ASTM D638-14, **Right**: Direction of printing for the compression test specimen according to Standard ASTM D695-15.

Due to the low cost of 3D-printers and greater availability of printers, we chose FDM printing technology. Specimens were printed using a Creality CR-10 S4 printer with a PLA filament with the commercial name PLA Original (AzureFilm, Sežana, Slovenia). An Ultimaker Cura 5.1.1. slicer was used to generate a G-code for 3D-printing. A 100% infill in the longitudinal and transverse directions was set, and the specimens were printed with a nozzle of 0.4 mm, layer height of 0.2 mm, print speed of 55 mm/s, bed temperature of 55 °C, and nozzle temperature of 210 °C, as recommended by the manufacturer. For tensile strength, the geometry of a Type IV specimen was chosen, in accordance with the Standard ASTM D638-14 (Figure 1—left). For the compression test, prisms of 6.35 mm × 6.35 mm × 12.7 mm were chosen in accordance with the ASTM D695-15 (Figure 1—right).

Measurements of the tensile properties of 3D-printed PLA specimens were performed using the Tinius Olsen testing machine H10KT (Tinius Olsen Ltd., Redhill, United Kingdom), following the Standard ASTM D638-14. Five Type IV molded specimens of 3D-printed PLA normal to (denoted as the longitudinal direction) and five parallel with the principal axis of anisotropy (denoted as the transverse direction) were prepared and conditioned in accordance with Procedure A of the ASTM D618-13 Standard (40/23/50) prior to testing. The width and thickness of each specimen were first measured according to test method B in the Standard ASTM D5947-11 using the Mitutoyo digital micrometer caliper at the same temperature and humidity used for conditioning, and then clipped to the tensile machine under the following conditions: speed of testing—50 mm/min, gauge length—65 mm. The load (N), elongation (mm, %) and strength (MPa) at break and yield points, and modulus of elasticity were recorded for all samples, and then the arithmetic mean and Standard Deviation were calculated and reported for each series of tests. Measurements of the compressive properties of plastics were also performed using the Tinius Olsen testing machine H10KT, following the Standard ASTM D695-15. Five specimens of 3D-printed PLA plastics normal to (denoted as the longitudinal direction) and five parallel with the principal axis of anisotropy (denoted as the transverse direction) were prepared and conditioned in accordance with Procedure A of the ASTM D618-13 Standard (40/23/50) prior to testing. The width and thickness of each specimen were measured first using the Mitutoyo digital micrometer caliper (Mitutoyo Corporation, Tokyo, Japan) at the same temperature and humidity used for conditioning, and then placed between the surfaces of the compression tool under the following conditions: speed of testing—1.3 mm/min.

The detailed results report encompassing uniaxial tensile and compression measurements performed on 3D-printed PLA material using the described methodology is available in the Supplementary Materials.

### 2.2. Numerical Model

#### 2.2.1. Geometry

Numerical analysis was performed using a previously developed generic transtibial model with an associated Patellar Tendon Bearing (PTB) socket and silicone liner [34]. The model represents an average male residual limb amputated according to surgical guidelines. The biological tissues, such as skin, subcutaneous tissue, and fat, were combined into a bulk soft tissue reflecting a global response, and adjusted according to the average male thigh, mid-patella, and calf circumferences.

**Figure 2:**
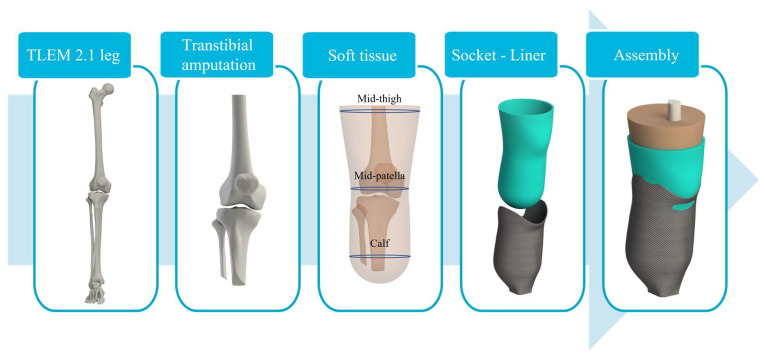
Modeling process of the generic numerical model geometry for the transtibial socket evaluation.

The generic residual limb model was fitted with a socket shaped according to the well-established PTB rectification method. PTB sockets tend to distribute the load to the more resilient areas, such as the patellar tendon, and relieve the pain-sensitive areas. In addition, a commonly used silicone liner was added to the model to reduce the stress concentration in the limb further, improving comfort for the amputee. The complete model can be seen in Figure 2, containing truncated and beveled bones (femur, tibia, patella, and fibula), bulk soft tissue, silicone liner, and the PTB socket.

#### 2.2.2. Material Models

The results of the experimental tests performed on the PLA dumbbell samples were averaged and used as the input for the numerical material model of the 3D-printed socket (Table 1). The PLA material responded linearly up to a yield point, followed by rapid fracture. Although the stiffness of the 3D-printed material is anisotropic, the Young’s modulus is similar in the longitudinal and transverse printing directions; therefore, the homogeneous isotropic linear-elastic material model was used (E = 2952.8 MPa, ν = 0.33). The anisotropy of the material was taken into account later in the evaluation phase, as the stress at the yield point differs significantly with respect to the printing direction. In order to evaluate the applicability of the biodegradable PLA for the prosthetic socket numerically, the 3D-printed material was compared with the frequently used materials PS (check socket) and composite (definitive socket). All transtibial socket models were defined using the linear-elastic material model with a specified Young’s modulus and Poisson’s ratio. The data for the material model of the definitive socket were taken from the study conducted at the University Rehabilitation Institute SOČA in Slovenia, where samples of composite materials used commonly for definitive sockets were tested and reported on [40]. The data for the PS, a polymer well-known in prosthetic practice for the fabrication of check sockets and well-suited for a rapid shape modification in situ, were taken from the literature (Table 2) [41].

The soft tissue responds non-linearly to compressive loading, and exhibits low stiffness at small strains, but the stiffness increases rapidly with increasing deformation. This property can be approximated in the simulation by the hyperelastic material model, which provides a more realistic behavior of the residual limb. Silicone liners react in a similar non-linear and incompressible manner, so the hyperelastic model can also define silicone liners. In the simulation, the 1st-order Ogden and the 3rd-order Yeoh material model were used for the bulk soft tissue and the silicone liner, respectively [42,43]. More detailed descriptions of the models can be found in the published study [34]. As the main object of the evaluation was the prosthetic socket, all supporting structures, such as the feet, the prosthesis rod, and the adapter, were modeled as rigid connections between the remote points.

#### 2.2.3. Boundary Conditions

**Figure 3:**
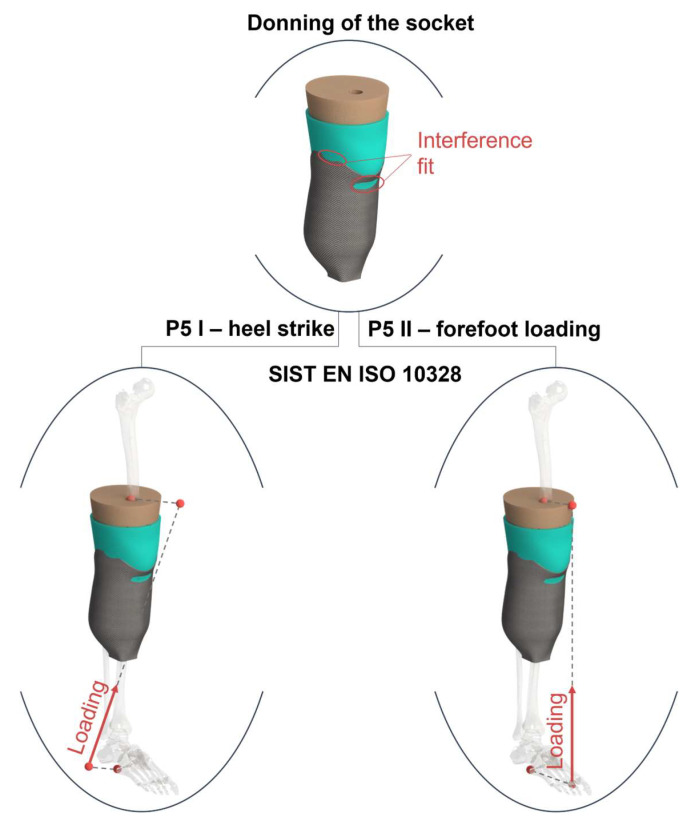
Load cases in the numerical simulation. **Upper**: Donning of the socket; the distal part of the socket and femoral cavity are fixed via remote points. **Left**: Heel strike loading case according to ISO 10328 P5 I. **Right**: Forefoot loading case according to ISO 10328 P5 II. Both Standard load cases were analyzed for the settling and proof test force. The red dots in the Figure represent remote points connected with rigid wires.

In order to prove the applicability of 3D-printed bio-based sockets numerically, various loading scenarios should be investigated that occur in daily use. Critical instances during the gait cycle should also be examined in addition to the donning of the socket, after which the initial stress state occurs inside the socket that influences the results [44]. To address this issue, heel strike and push-off conditions were simulated according to ISO 10328. Applying the Standard test procedure in a virtual environment, an important step is to include the crucial loading cases that occur during the gait cycle. The static tests in the Standard are divided into P-levels, depending on the weight of the lower-limb amputee. The numerical model used represents an average male weighing 85.6 kg according to the National Health Statistic Reports 2008, which fits into the P5 level [45]. The static P5 loading conditions were tested for both heel strike and push-off, with two different loading intensities: Settling Test Force and Proof Test Force, corresponding to the loads experienced during normal gait, and occasional severe events such as tripping or falling, respectively. All loading conditions are described and presented graphically in Table 3 and Figure 3.

Donning of the prosthesis was simulated by solving the initial interference between the socket and the residual limb, while fixing the attachment plane of the adapter and the femoral cavity. Due to the significant difference in stiffness between the bones and the soft tissue, the bones can be modeled as a rigid structure, as their deformation is negligible compared to the tissue. Therefore, the bone cavities within the soft tissue were modeled as rigid surfaces that limited the bones’ deformation and connected them to the soft tissue. The silicone liner has a high coefficient of friction (CoF) (more than 2) when interacting with the skin [46]. Therefore, a rough contact between the silicone liner and the soft tissue was defined, allowing for separation in the normal direction, and preventing tangential slippage. Silicone liners usually have a fabric coating with a lower CoF when in contact with the composite or polymer material. Hence, a CoF of 0.5 was set between the liner and the socket, as reported in the literature [47].

## 3. Results

The numerical simulation was calculated on an HPC-core at the University of Maribor using ANSYS Solver. The HPC ran on a LINUX x64 platform with an Intel^®^ Xeon^®^ CPU E5-2670 0 processor with 16 cores. The average CP times, depending on the type of socket material for the load cases P5 I and P5 II, were 14 h 9 min and 19 h 49 min, respectively.

### 3.1. PLA Socket Strength Test

**Figure 4:**
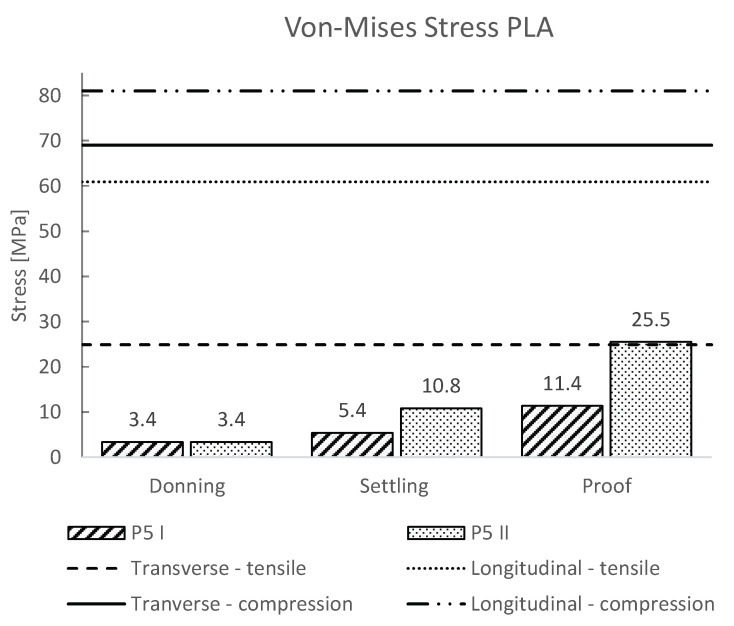
Von-Mises stress of the 3D-printed PLA socket for the loading cases P5 I and P5 II. Additionally, the transverse and longitudinal yield stresses are shown with horizontal dashed lines.

**Figure 5:**
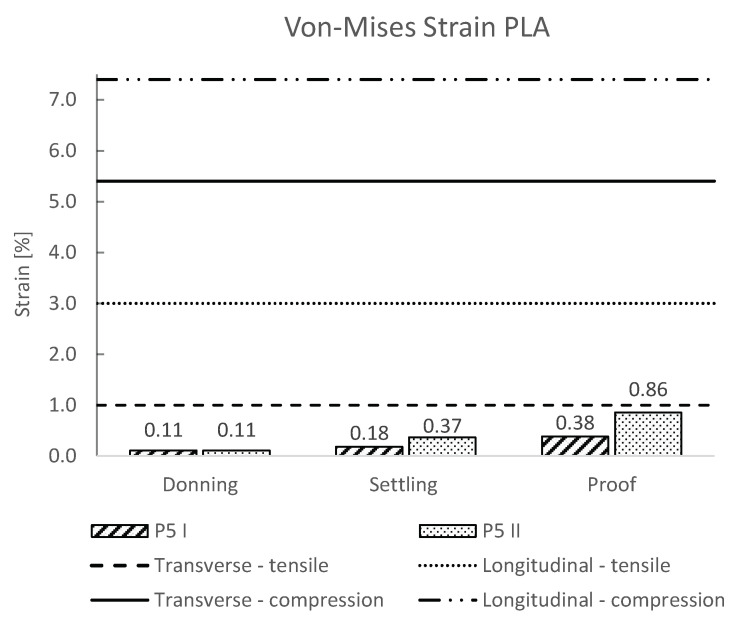
Von-Mises strain of the 3D-printed PLA socket for the loading cases P5 I and P5 II. Additionally, the transverse and longitudinal yield strains are shown with horizontal dashed lines.

The maximum values of Von-Mises stresses and strains for all load steps were extracted from the numerical calculations, and are presented in Figure 4 and Figure 5, respectively. In addition to the numerical results, the compressive and tensile yield stress/strain for the transversely and longitudinally 3D-printed PLA samples were included in the plot.

**Figure 6:**
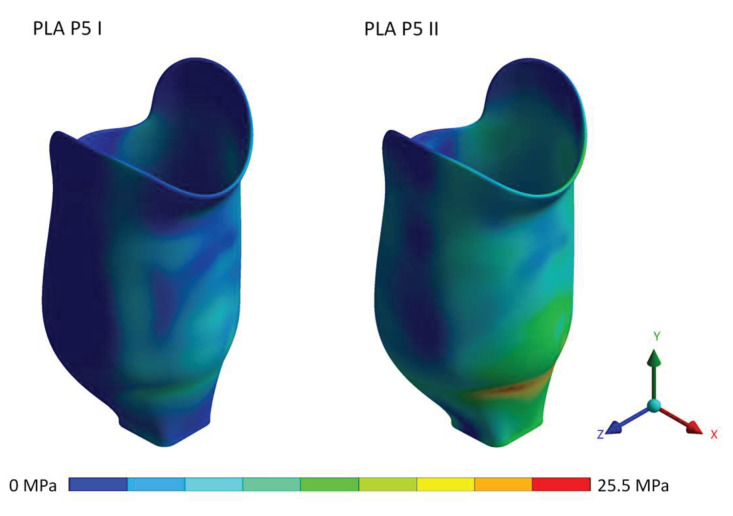
Von-Mises stress distribution for the PLA socket at proof test force.

Figure 6 shows the Von-Mises stress for heel strike and push-off loading conditions at proof test force, displaying the stress distribution in the socket.

**Figure 7:**
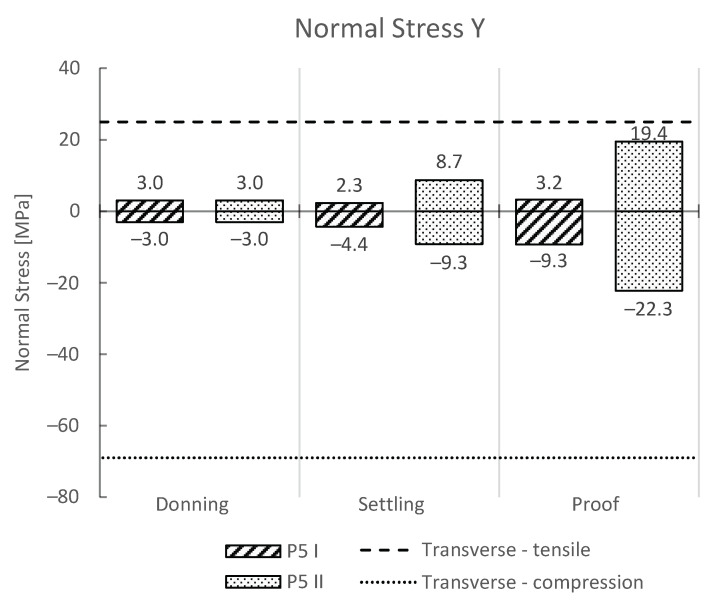
Results of normal stress in the y-axis. The negative values represent compression, while the positive values represent tensile stress. Additionally, the transverse yield stress is shown with horizontal dashed lines.

**Figure 8:**
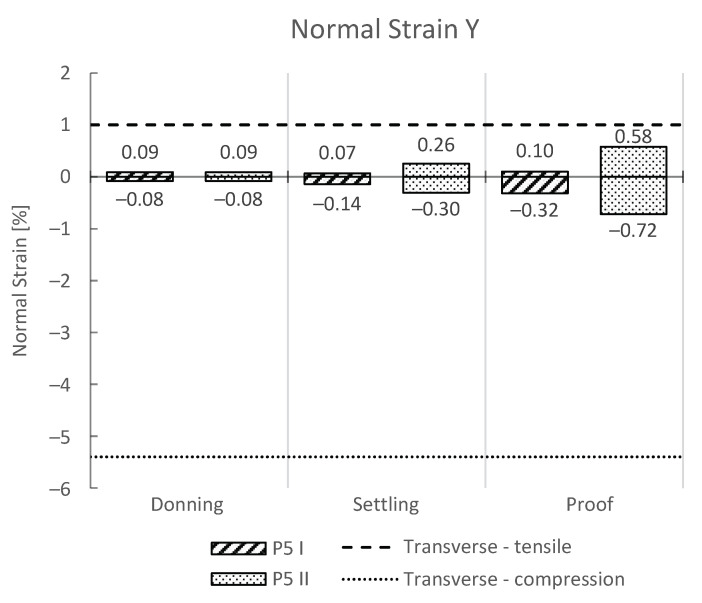
Results of normal strain in the y-axis. The negative values represent compression, while the positive values represent tensile strain. Additionally, the transverse yield strain is shown with horizontal dashed lines.

As a common problem during mechanical loading of 3D-printed parts is the delamination of the layers, we also provide the results of maximum normal stress and strain in the vertical direction (y-axis) for the donning, settling, and proof load steps (Figure 7 and Figure 8, respectively). The yield stresses and strains obtained from uniaxial tensile and compression tests for transversely 3D-printed PLA samples were added to the plots.

**Figure 9:**
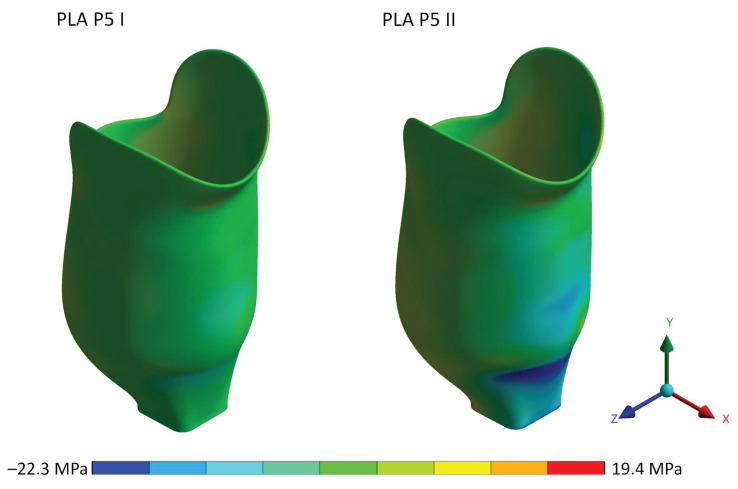
Normal stress distribution in the y-axis for a PLA socket at proof test force. Negative values stand for compressive stress, positive values for tensile stress.

Figure 9 shows the normal stress in the y-axis for heel strike and push-off loading conditions at proof test force, displaying the normal stress distribution in the socket.

Table 4 summarizes the stress and strain results from the numerical simulation for all load levels.

### 3.2. Biomechanical Analysis of Transtibial Sockets

**Figure 10:**
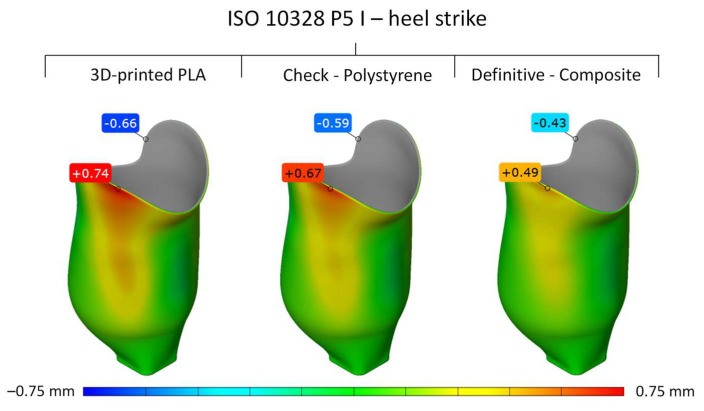
Global deformation of PLA, composite, and polystyrene sockets for load case P5 I at settling test force.

**Figure 11:**
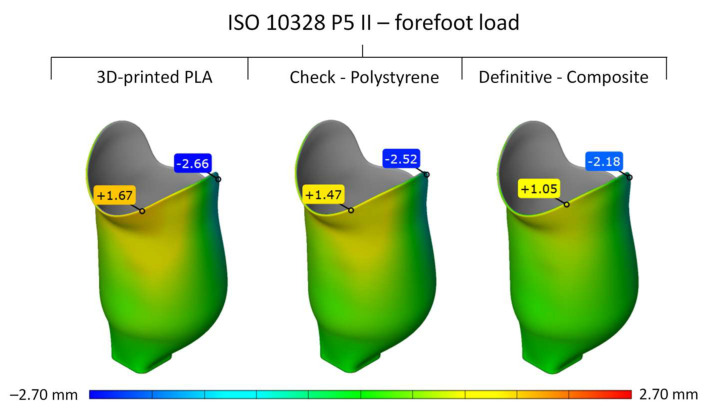
Global deformation of PLA, composite, and polystyrene sockets for load case P5 II at settling test force.

The deformed sockets, after numerical simulation, were exported from the ANSYS software and compared to the original socket shape in the GomInspect (Zeiss, Germany) to obtain the relative deformation for the given load case. Figure 10 and Figure 11 show the global deformation of the outer surface of the PLA, composite, and polystyrene sockets in mm for both load cases at settling test forces, where the positive values represent the surface deformation out of the original surface (red color), and negative values represent the surface deformation inside of the original surface (blue color).

Table 5 provides an overview of the global deformation results obtained from the numerical simulation at the settling test force for the 3D-printed PLA, polystyrene, and composite socket.

## 4. Discussion

### 4.1. PLA Socket Strength Test

To explore the possibility of utilizing non-engineering PLA plastic and the technology of FDM 3D-printing in the development and manufacturing of lower-limb prosthesis sockets, the primary goal of this research was to check whether the 3D-printed socket using PLA could withstand the most typical static loading scenarios during normal gait according to ISO 10328.

**Figure 12:**
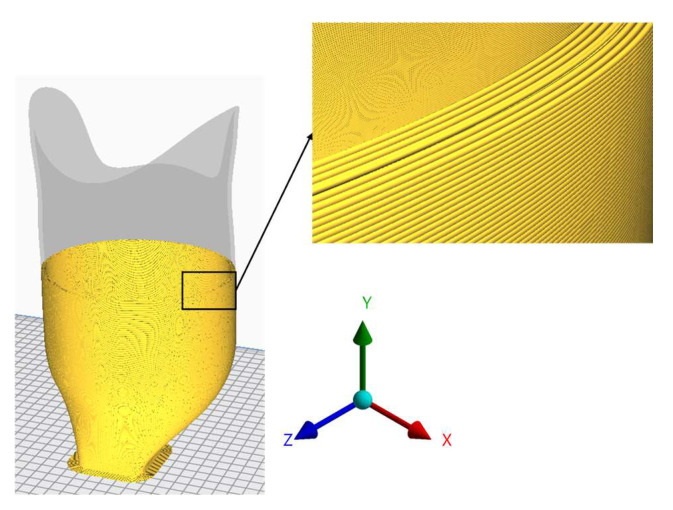
Printing setup with coordinate system—the printing direction follows the y-axis.

Experimental strength testing and comparison to the previous research showed that 3D-printed PLA specimens can withstand considerably lower values of mechanical stress compared to the composite materials used commonly for definitive prosthesis sockets. Additionally, the stress values at a yield of 3D-printed PLA specimens were dependent on the printing direction, and also the direction of the loading (compression and tension). The presented anisotropy of the material properties and different yield values for compression and tension were expected, and they are a known phenomenon occurring due to the FDM 3D-printing technology as presented in the introduction. Hence, in transversely loaded specimens, the weakest link is the resistance of the union between layers. This means that the 3D-printing direction and, therefore, orientation of the filament layers must be coincident with the predominant stresses in the 3D-printed part for maximum strength. This is not always possible, due to restrictions of print volume of the most common FDM 3D-printers, the part size, and geometrical features of the part. To be able to 3D-print the prosthesis socket without support material, the prosthesis socket model was oriented in a way that the flat socket adapter would be printed first, and the remaining socket would be printed standing up (Figure 12).

The results from the numerical simulations showed that donning resulted in a 3.4 MPa von-Mises stress and 0.11 Strain (Figure 4 and Figure 5). Both values are way lower than the yield and maximum levels of strain of the PLA for both directions of loading (Table 1). This was expected, as the stresses and strains at the donning of the prosthesis are the result of the rectification of the socket that distributes the contact pressure to the preferred anatomical areas with low sensibility. In this manner, loads from the patient’s limb and reaction forces from the ground can be transferred effectively to the stump for the most comfortable fit with appropriate prosthesis stability.

The results of the von-Mises stress and strain of the socket during the gait cycle (settling force) for heel strike (P5 I) and forefoot loading (P5 II) according to ISO 10328 showed that forefoot loading resulted in a higher value of von-Mises stress of 10.8 MPa compared to a heel strike of 5.4 MPa (Figure 4). The obtained results are way lower than the yield values of PLA for both directions of 3D-printing and are also lower than the maximum strain values (Table 1). Hence, a safety factor of at least 2 was obtained, which confirmed that the 3D-printed socket made from PLA plastic would withstand the stresses and strains during a normal gait cycle. This has also been confirmed with an observational study by the previous research of van der Stelt et al. [17].

Additionally, in our numerical simulations, we also considered the proof test force according to ISO 10328, representing an occasional severe event such as a trip or fall, where the prosthesis is loaded way past the normal loading scenario during the gait cycle. The results showed that the von-Mises stress occurring in the socket during forefoot loading (P5 II) exceeded the yield values for the PLA in the transverse direction of printing slightly (Figure 4). The values for the heel strike remained below the yield value of the PLA in both printing directions. The obtained values of strain also remained below the yield strain (Figure 5).

Von-Mises stress/strain includes all types of stresses, provides a good general insight into the stress/strain distribution in the model, and is particularly useful when evaluating complex loading scenarios and homogeneous materials, such as metals. Experimental testing showed that the mechanical properties of the 3D-printed PLA specimens and parts are strongly dependent on the direction of printing, and also on the direction of the stress (compression and tension); therefore, the von-Mises yield criterion can only be used for the initial analysis.

Human gait using a lower-limb prosthesis results in highly dynamic loading; however, research has shown that the highest stresses and strains occur in the socket in the transverse direction (y-axis) (Figure 7 and Figure 8). Therefore, additionally to the von-Mises criterion, we also analyzed the stresses and strains in the y-axis. The results from the numerical tests have confirmed that the maximum contribution to the von-Mises stress are the tensile and compressive stresses in the y-axis, as has been reported previously. Therefore, the maximum stresses in the y-axis were compared to the stress and strain yield values of the PLA in the transverse direction for compression and tension (Figure 7 and Figure 8). Both the donning and settling test force for load cases of heel strike and forefoot loading resulted in stresses and strains in the y-axis, which are considerably lower than the maximum yield stress and strain for the PLA in the transverse direction. A considerable difference in maximum stress and strain values was observed between heel strike and forefoot loading, where forefoot loading showed higher values of stress and strain values by two times or even more (Table 4). The difference was especially evident in the tensile direction and can be attributed to a larger moment arm of the force vector (Figure 7). Even higher values of stress and strain were observed for the proof test force. The highest value of tensile stress was observed in the y-axis with forefoot loading with 19.4 MPa, and was closest to the reported tensile stress yield value of 24.9 MPa for the 3D-printed PLA in the transverse direction (Figure 7). The strain values were lower, and way below the yield strain value.

To be able to perform relative analysis between sockets with different materials, all sockets in the numerical analysis had the same geometry and thickness. While the obtained result of maximum stress in the y-axis for the forefoot loading case was still lower than the yield value of the 3D-printed PLA in the transverse direction, the final 3D-printed socket from PLA should be modified to include thicker walls for lowering the maximum stress values, to be able to obtain a suitable factor of safety.

In summary, the results from the numerical analysis showed that the 3D-printed PLA prosthesis socket with the same geometry and wall thickness as a check PS socket and definitive composite socket would withstand the donning, settling, and proof test forces listed in ISO 10328 without plastic deformation, or even teardown.

### 4.2. Biomechanical Analysis of Transtibial Sockets

The rectification process of the prosthesis creates a preloading on the stump for the effective transfer of forces from the stump to the prosthesis and the ground, and vice versa. Therefore, the soft tissue (skin, subcutaneous tissue, muscles, etc.) is deformed and, hence, preloaded with the modified shape of the socket. Due to the preloading, the comfort of the prosthesis fit is lowered. In this regard, the preloading of the stump is usually a compromise of prosthesis comfort and stability. To maximize stability, the prosthesis socket should deform elastically as little as possible as a result of stresses on the socket due to the gait cycle.

Hence, after confirming that the proposed 3D-printed PLA prosthesis socket can withstand all loading scenarios, the secondary goal of the numerical simulation was to analyze the effect of the material on the prosthesis’ stability during the gait cycle (forefoot load and heel strike load), and during the occasional severe event of proof test force according to ISO 10328.

Excessive deformation of the socket may compromise the stability of the prosthesis, so minimal deformation is desirable. Previous research has shown that the perceived stability of the prosthesis by the user is highly subjective; therefore, no definitive values of maximum allowed socket elastic deformation have been provided by the authors. Hence, the relative comparison and analysis of different materials and resulting displacements need to be performed and compared to the commonly used composite socket. Therefore, we extracted the results of displacements for the settling test force for heel strike as well as forefoot loading for all materials.

The results from the numerical simulations have shown that the highest deformations and, hence, displacements of the socket were obtained by the upper part of the socket, as shown in Figure 10 and Figure 11. This was expected, as this is the part of the socket that is furthest away from the mounting of the artificial leg. The moment arm during the distinctive load cases of the heel strike and forefoot stance is greatest, which results in the highest loads on the socket, with the highest deformations and displacements.

The results for the heel strike have shown that the socket made from the composite commonly found in prosthetics resulted in the lowest displacements of 0.49 mm when compared to the original 3D CAD socket model (Figure 10). The polystyrene socket, which is used mostly as a test socket, resulted in a slightly higher maximum displacement of 0.67 mm, and the socket made from PLA resulted in 0.74 mm of displacement.

The forefoot loading case resulted in higher stresses due to a larger moment arm; therefore, higher elastic deformations and displacements were also expected. This was also confirmed where the composite socket showed the highest displacement of −2.18 mm. Once again, the polystyrene socket showed a maximum displacement of the socket of −2.52 mm, and PLA showed −2.66 mm (Figure 11).

Based on previous studies, subjects reported that the polystyrene socket provided the same subjective perceived stability as the final composite socket. As polystyrene and PLA show a similar modulus of elasticity, the resulting displacements are also very similar, and show only a small difference for all the considered load cases. The difference was even smaller when compared to the definitive socket of composite material. Hence, it can be concluded that the proposed PLA socket would provide the same perceived subjective stability as the polystyrene socket for both load cases.

This study is a preliminary numerical study, which has shown that a 3D-printed bio-based PLA can withstand the most distinctive loads during the gait cycle, and can provide the same stability as a common composite prosthesis socket. However, human walking is a dynamic process; therefore, dynamic simulations should be performed for future work. Additionally, we only considered static material strength values. Future work should, therefore, also consider fatigue failure mechanisms to analyze if the stress levels are below the fatigue limit. The numerical simulations could be expanded further to perform topological optimization of the socket geometry for minimizing the stresses and displacements, as 3D-printing technology allows the manufacturing of exact and complex geometries. Finally, a 3D-printed bio-based PLA socket should be manufactured, tested experimentally, and analyzed using test subjects.

## 5. Conclusions

A newly developed and biomechanically validated generic numerical model of a transtibial-limb–socket system was utilized to simulate and analyze whether a prosthesis socket manufactured using a low-cost FDM 3D-printer and non-engineering biodegradable PLA material filament can be used as an alternative to common composite sockets. In this study, we expanded on the boundary conditions according to ISO 10328, where, additionally to the donning phase, a heel strike and forefoot loading were considered, resulting in realistic mechanical loads on the socket, which made the evaluation and analysis of different socket materials possible. The results showed that the proposed 3D-printed PLA socket would withstand all loads during the donning phase, forefoot loading, and heel strike according to ISO 10328 (settling and proof test), therefore making the 3D-printed PLA socket functional and safe to use. The results also showed that the 3D-printed PLA socket would result in similar displacements to check sockets usually made from polystyrene, therefore maintaining subjective perceived stability. In this study, we have shown that the newly developed generic numerical model of a transtibial-limb–socket system can be used successfully to analyze various design choices of prosthesis sockets, such as materials inside the virtual environment, providing a tool for more integrated CAx socket development. Due to the needed frequent changes of prostheses, sockets made using computer-aided technologies and biodegradable materials such as PLA would result in significant reductions in time and cost.

## Figures and Tables

**Table 1:** Yield stress and strain for 3D-printed PLA material determined by uniaxial tensile and compression tests.

	Yield Strain (%)		Yield Stress (MPa)	
	Tensile	Compression	Tensile	Compression
PLA longitudinal	3	7.4	60.9	81
PLA transverse	1	5.4	24.9	69

**Table 2:** Material properties for composite and polystyrene sockets used in the numerical simulation.

	Yield Strain (%)	Yield Stress (MPa)	E (MPa)	Poisson’s Ratio
Composite	N/A	193	4991	0.3
Polystyrene	1.4	48	3400	0.34

**Table 3:** Summary of loading cases with corresponding loads.

Loading Case	Donning of the Socket	ISO P5 I (Heel Strike)		ISO P5 II (Push-Off)	
		Settling Force	Proof Force	Settling Force	Proof Force
Load (N)	Interference fit	1024	2240	920	2013

**Table 4:** Summary of maximum stress and stain numerical results for all loading conditions.

	Donning		Settling		Proof	
	P5 I	P5 II	P5 I	P5 II	P5 I	P5 II
Von-Mises stress (MPa)	3.4	3.4	5.4	10.8	11.4	25.5
Tensile stress (y-axis) (MPa)	3.0	3.0	2.3	8.7	3.2	19.4
Compression stress (y-axis) (MPa)	3.0	3.0	4.4	9.3	9.3	22.3
Von-Mises strain (%)	0.11	0.11	0.18	0.37	0.38	0.86
Tensile strain (y-axis) (%)	0.09	0.09	0.07	0.26	0.10	0.58
Compression strain (y-axis) (%)	0.08	0.08	0.14	0.30	0.32	0.72

**Table 5:** Summary of maximum deformation results obtained by numerical simulation at settling test force for PLA, polystyrene, and composite sockets.

Global Deformation (mm)	3D-Printed PLA	Polystyrene	Composite
P5 I—settling test force	0.74	0.67	0.49
P5 II—settling test force	2.66	2.52	2.18

## Data Availability

The data presented in this study are openly available in Uniaxial test report 2.1 at Supplementary Materials.

## Supplementary Materials

The following supporting information can be downloaded at: https://www.mdpi.com/article/10.3390/ma16051985/s1, Uniaxial test report 2.1.
